# Are both sympatric species *Ilex perado *and *Ilex canariensis *secretly hybridizing? Indication from nuclear markers collected in Tenerife

**DOI:** 10.1186/1471-2148-4-46

**Published:** 2004-11-18

**Authors:** Jean-François Manen

**Affiliations:** 1 Université de Genève, Conservatoire et Jardin Botaniques, Impératrice 1, CH-1292 Chambésy/Genève, Switzerland

## Abstract

**Background:**

Intra-specific and intra-individual polymorphism is frequently observed in nuclear markers of *Ilex *(Aquifoliaceae) and discrepancy between plastid and nuclear phylogenies is the rule in this genus. These observations suggest that inter-specific plastid or/and nuclear introgression played an important role in the process of evolution of *Ilex*. With the aim of a precise understanding of the evolution of this genus, two distantly related sympatric species collected in Tenerife (Canary Islands), *I. perado *and *I. canariensis*, were studied in detail. Introgression between these two species was previously never reported. One plastid marker (the *atpB-rbcL *spacer) and two nuclear markers, the ribosomal internal transcribed spacer (ITS) and the nuclear encoded plastid glutamine synthetase (*nepGS*) were analyzed for 13 and 27 individuals of *I. perado *and *I. canariensis*, respectively.

**Results:**

The plastid marker is intra-specifically constant and correlated with species identity. On the other hand, whereas the nuclear markers are conserved in *I. perado*, they are highly polymorphic in *I. canariensis*. The presence of pseudogenes and recombination in ITS sequences of *I. canariensis *explain this polymorphism. Ancestral sequence polymorphism with incomplete lineage sorting, or past or recent hybridization with an unknown species could explain this polymorphism, not resolved by concerted evolution. However, as already reported for many other plants, past or recent introgression of an alien genotype seem the most probable explanation for such a tremendous polymorphism.

**Conclusions:**

Data do not allow the determination with certitude of the putative species introgressing *I. canariensis*, but *I. perado *is suspected. The introgression would be unilateral, with *I. perado *as the male donor, and the paternal sequences would be rapidly converted in highly divergent and consequently unidentifiable pseudogenes. At least, this study allows the establishment of precautionary measures when nuclear markers are used in phylogenetic studies of genera having experienced introgression such as the genus *Ilex*.

## Background

Aquifoliaceae comprise one genus, *Ilex *[1] and approximately 400 species. The fossil record indicates that the genus was cosmopolitan during the Eocene. It is now largely extinct in Australia, Europe and Africa where only few species persist. Most diversity is currently found in South-America and in Southeast-Asia. They are evergreen or deciduous trees or bushes living in warm-moist-temperate, sub-tropical, tropical or montane-tropical areas.

The molecular phylogeny of the genus *Ilex *[2,3] shows that systematic relationships are still not well understood. The plastid phylogeny (inferred from the *atpB-rbcL *spacer, *rbcL *and *trnL-F*) is highly correlated with the geographic distribution of extant species. Four chloroplast clades are found: one exclusively Eurasian clade, one exclusively American clade and two different North-American/Asian clades (one of them comprising most of the deciduous species among other evergreen species). On the other hand, the nuclear phylogeny (inferred from ribosomal ITS and the 5S RNA spacer) is incongruent with the plastid phylogeny, suggesting frequent interlineage hybridizations. The nuclear phylogeny is not correlated with the geographic distribution of extant species.

Any of the plastid or the nuclear phylogeny corroborates previous morphological or biosystematic studies [3]. Using chloroplast RFLPs, *trnL-trnF *sequencing and nuclear ITS sequencing, a study of Asian *Ilex *of the Bonin Island and of the Ryukyu Island [4] confirmed that hybridization played a role in this region, leading to interspecific introgressions independently observed on both Islands. RAPD data indicate that the Japanese species *Ilex leucoclada *M. is highly polymorphic [5]. During its history, the genus *Ilex *probably experienced frequent incomplete lineage sorting and nuclear and/or cytoplasmic introgression, making the study of its history very complex.

Few data are reported on the chromosome number of *Ilex *[6]. The basic haploid number is 20, with deviation to 17, 18 and 19. From the 27 chromosome numbers available for the genus *Ilex*, three species are tetraploid (*I. anomala, I. verticillata *and *I. argentina*) and one species is hexaploid (*I. pedunculosa*), indicating probable hybridizations between species having divergent genomic background (alloploidy).

A previous study [3] showed that individuals of many species of *Ilex *contain polymorphic nuclear sequences (ITS and 5S rDNA spacer). Except for *I. purpurea *and *I. guianensis*, only one individual was studied per species. The sampling being too low for a correct evaluation of this intraspecific polymorphism, an exhaustive study of one plastid marker (the *atpB-rbcL *spacer) and two nuclear markers (the ribosomal internal transcribed spacers, ITS, and the nuclear encoded plastid glutamine synthetase, *nepGS*) was undertaken on several individuals of *I. perado *and *I. canariensis *collected in Tenerife (Canary Islands). These species were chosen because, based on DNA data, they are not closely related [2,3] and are growing sympatrically in Canary Islands. Both species are morphologically variable but few characters allow species identification. *I. canariensis *is endemic of Canary Islands, whereas *I. perado *has a wider distribution in Spain, Portugal, North-Africa and Canary Islands. Natural or artificial hybridization between both species was never reported. The data show that, contrarily to *I. perado*, *I. canariensis *has highly polymorphic ITS and *nepGS *sequences. The aim of this study was to (1) explain the polymorphism observed in ITS of *I. canariensis *by an investigation of its pattern of substitution and its functionality, (2) determine the evolutionary mechanisms responsible of this polymorphism and (3) focus on ITS evolution and consequences for phylogenetic reconstruction of the genus *Ilex*.

## Results

### ITS polymorphism

**Figure 1:**
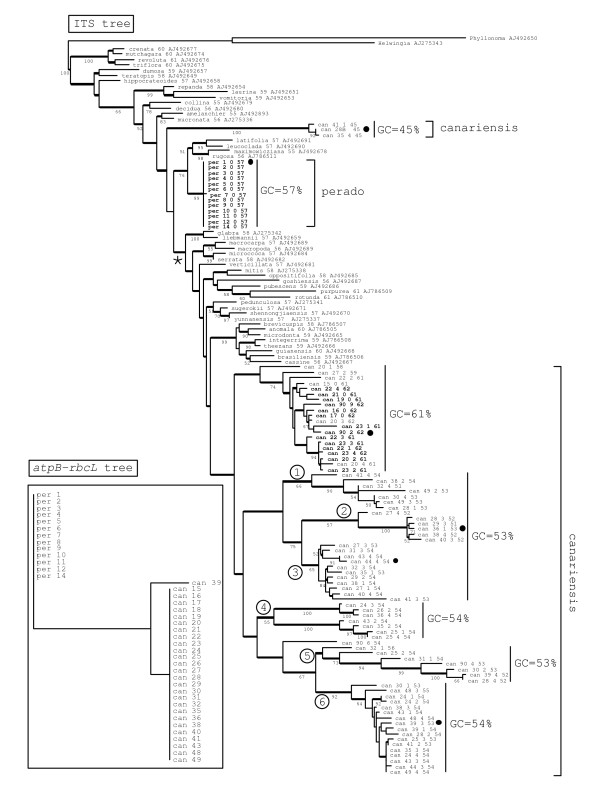
Most parsimonious atpB-rbcL phylogram (in the inset) and one of the most parsimonious ITS phylogram. Branches conserved in the strict consensus ITS tree are thicker. Bootstrap values are indicated bellow the branches. ITS sequences of 43 *Ilex *species [3] are indicated by their species name followed by their GC content (in %) and by their DNA accession code in GenBank. ITS sequences of *I. perado *and *I. canariensis *collected in Tenerife are indicated by a species code ("per" and "can") and 3 numbers: "can 41_1_45" means *I. canariensis *specimen 41, clone 1, with a GC content of 45%. Clone 0 means that the sequence was read directly from the PCR product. Specimen "can 28B" represents the shorter PCR product found in specimen 28 of *I. canariensis*, cut out from agarose gel (see Figure 3). The average GC content is indicated for each clades of the ITS tree. Circled numbers refer to clades discussed in the text (see Figure 4). Bold characters indicate ITS sequences with no substitution at conserved 5.8S sites (see results). Black dots indicate ITS sequences studied in more details (see results). The asterisk indicates an alternative position of the GC 45% clade.

Figure 1 (inset) shows the unique plastid *atpB-rbcL *spacer phylogram obtained from the alignment of the sequences of *I. perado *and *I. canariensis *collected in Tenerife. All individuals of *I. perado *have the same *atpB-rbcL *spacer sequence. For *I. canariensis*, 26 individuals have the same *atpB-rbcL *spacer sequence and three substitutions are observed (in a T-rich variable region) for specimen 39. This plastid marker perfectly agrees with species determination.

As the ITS sequences found in *I. canariensis *are extremely polymorphic, it was interesting to observe their relationships with available ITS sequences previously investigated species by Manen et al. [3]. Figure 1 shows one of the most parsimonious ITS tree of *I. perado *and *I. canariensis *sequences found in Tenerife altogether with 43 ITS sequences of other *Ilex *species. Thick bars indicate internal branches conserved in the consensus tree. The closest possible outgroups for the genus *Ilex*, are *Helwingia *and *Phyllonoma*. However these genera are so isolated systematically that their use to root *Ilex *should be taken with care. On 13 individuals of *I. perado*, only one substitution (a transition) is observed in ITS. On the other hand, ITS sequences of most individuals of *I. canariensis *are polymorphic and few sequences are identical. The divergence between all ITS sequences observed in *I. canariensis *is much higher than between available ITS sequences of all other species investigated. The GC content is 57% for *I. perado *and from 45 to 62% for *I. canariensis*. Regarding their GC content, three groups of ITS are found in *I. canariensis*: a clade with 45% GC, a clade with 61% GC in average and several clades with 53–54% GC in average. The GC content of other investigated species range from 55 to 61% (Figure 1).

The ITS sequences of *I. canariensis *are distributed in two groups in the phylogram represented in Figure 1. One group forms a large clade conserved in the consensus tree but not sustained by bootstrap statistics. Another group forms a small clade (GC 45%) which branches variably: as indicated in Figure 1, or at the position indicated by the asterisk.

**Figure 2:**
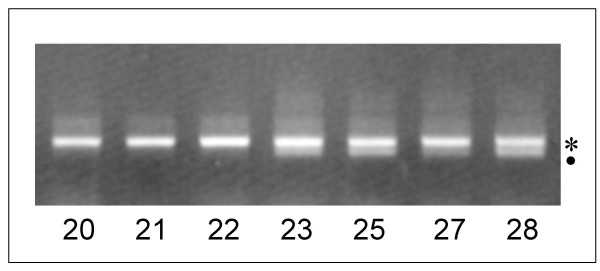
Example of agarose gel electrophoresis of ITS PCR products of individuals of *I. canariensis*. The line numbers represent individuals of *I. canariensis*. The star indicates the position of the expected functional ITS band and the dot indicates the position of the GC 45% ITS pseudogene band.

The sequences of the GC 45% clade have a 110 bp deletion in ITS 1 and are suggested to represent pseudogenes. In many ITS PCR products of individuals of *I. canariensis*, a shorter PCR band is visible on ethidium bromide agarose gel electrophoresis altogether with the main ITS band (Figure 2). In specimen 28, this electrophoretic band has been cut out and directly sequenced (sample "can 28B"). It has a sequence very close to both the cloned sequences of the GC 45% clade found in specimens 35 and 41. Thus these putative pseudogenes seem rather common in *I. canariensis*. During selection of the clones to be sequenced, the longest PCR fragments were favored with the aim to select functional sequences. Thus, as the shorter pseudogene band seems frequent in *I. canariensis*, Figure 1 underscores this class of ITS sequences represented by the clade GC 45%.

### Secondary structure of ITS 2

**Figure 3:**
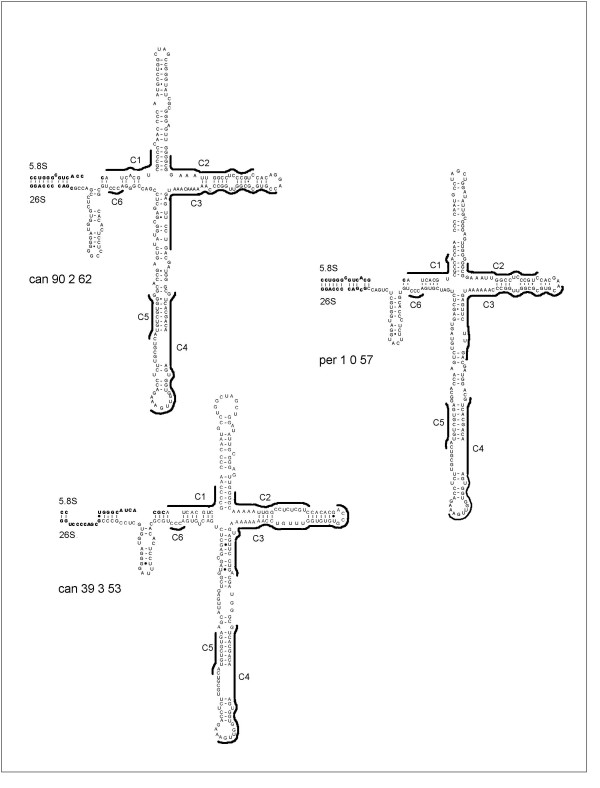
Functional secondary structures of some ITS 2 sequences of *I. perado *and *I. canariensis *according to Hershkovitz and Zimmer [7]. The flanking coding regions (3'end of 5.8S and 5'end of 25S) are indicated in bold characters. Conserved regions (C1 to C6) are indicated.

The secondary structure of both ITS regions is involved in the processing of the rRNA precursor and is thus constrained for this function. In angiosperms, an ITS 2 secondary structure has been proposed and comprises 6 conserved regions (C1 to C6) which are involved in common pairing relationships on the structure [7]. In order to determine which ITS sequences found in *I. canariensis *are functional, the secondary structure of ITS 2 was investigated from selected sequences representing a good sampling of ITS (sequences marked with a dot in Figure 1: per 1_0_57, can 28_B_45, can 36_1_53, can 44_4_54, can 90_2_62 and can 39 3 53). Figure 3 shows functional secondary structures found using Mfold [8]. Only the sequence of per 1_0_57, can 90_2_62 and can 39_3_53 provide an apparently functional ITS 2 secondary structure showing the common pairing relationships of conserved C1 to C6 regions according to Hershkovitz and Zimmer [7]. No such ITS 2 secondary structure was found for sequences can 28B_45, can 36_1_53 and can 44_4_54, suggesting pseudogenes.

### Pattern of substitution

The GC content, indicated for all ITS sequences of Figure 1, suggests that the pattern of substitutions is biased towards A or T for sequences of the GC 45% clade and for sequences of the GC 53–54% clades, as expected for pseudogenes. A reconstructed ancestral sequence of *Ilex *was calculated by maximum likelihood from the ITS data of Manen et al. [3] and used to investigate the pattern of substitution on selected ITS sequences. Table 1 shows that a higher rate of substitution is observed for ITS sequences found in the GC 45% and GC 53–54% clades than for the *I. perado *sequences and for the *I. canariensis *sequences found in the GC 62% clade. This increased rate is statistically significant according to the Kruskal-Wallis rank test [9].

**Table 1:** Substitution patterns of *I. perado *and *I. canariensis  *ITS sequences from a reconstructed maximum likelihood ancestral ITS sequence of *Ilex*.

	**Rate**	**nmC**	**mC**	**Chi2**
**per 1_0_57**	0.045	4/226 (1.77%)	11/174 (6.32%)	*
**can 28_B_45**	0.188	24/226 (10.62%)	37/174 (21.26%)	**
**can 39_3_53**	0.080	11/226 (4.87%)	19/174 (10.92%)	*
**can 44_4_54**	0.061	12/226 (5.31%)	16/174 (9.20%)	ns
**can 36_1_53**	0.090	10/226 (4.42%)	26/174 (14.94%	***
**can 90_2_62**	0.055	0/226 (0.00%)	2/174 (1.15%)	ns

Rate: Kimura 2-parameter distance from the reconstructed ancestral sequence. nmC: Number of C>T substitutions / number of non-methylated cytosines on both DNA strands of the reconstructed ancestral ITS sequence of *Ilex*. mC: Number of C>T substitutions / number of methylated cytosines on both DNA strands of the reconstructed ancestral ITS sequence of *Ilex*. The corresponding ratios of C>T substitutions are indicated between brackets. Chi2: Chi-square homogeneity test between expected and observed C>T substitutions at methylated cytosines (ns: non significant; *, ** and ***: significant at 0.05, 0.01 and 0.001 levels, respectively).

As expected for pseudogenes [10,11], the observed rate of deamination-like substitutions at methylated cytosine sites (CpG and CpNpG sites) is higher than the expected rate of C -> T and G -> A substitutions at non-methylated sites for can 28B_45, can 39_3_53, can 44_4_54 and can 36_1_62 (Table 1). A chi-square homogeneity test [9] indicates that this is highly significant for can 28B_45 and can 36_1_53, which certainly represent pseudogenes.

### Substitutions at conserved sites of the 5.8S rDNA

The alignment of fifty 5.8S sequences (modified from Muir et al. [11]) shows that 59 sites are conserved in vertebrates, invertebrates, fungi and plants and are expected to be functionally constrained. Substitutions observed at these sites would suggest non-functional pseudogenes. Contrarily to *I. perado*, many ITS sequences found in *I. canariensis *have substitutions at some of these conserved sites. Only the GC 61% clade of *I. canariensis *comprises non-substituted conserved 5.8S sites (sequences indicated in bold in Figure 1). Sequences of the GC 45% clade have 10–11 substitutions. Sequences of the GC 53–54% clades have 2 to 7 substitutions. Three sequences of the GC 61% clade have only one mutation, which may be PCR artifacts [11] and two sequences (can_20_1_58 and can_27_2_59) with a lower GC content (58 and 59%, respectively) have 2 mutations. Thus ITS sequences of *I. canariensis *having a GC content higher than 60% are expected to be functional genes, all other sequences with lower GC content are suspected to be pseudogenes.

### Recombinations

**Figure 4:**
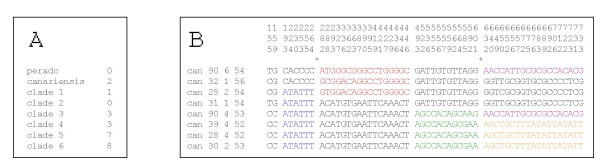
Recombination evidence in ITS sequences. A: Minimum number of recombination events in ITS clades (numbered as in Figure 1) calculated using the DnaSP program [12]. "perado": *I. perado *clade. "canariensis": GC 61% clade of *I. canariensis *representing functional ITS sequences. B: An example of obvious recombined ITS sequences found in *I. canariensis *clade 5. Only informative nucleotides are represented. Homologous sequence fragments have the same color. Stars indicate the recombination points found by maximum likelihood (program LARD) for sequences can 90_6_54, can 25_2_54 and can 90_4_53 (see results).

Most ITS sequences of *I. canariensis *experienced frequent recombinations: in the entire ITS matrix of *I perado *and *I. canariensis*, the DnaSP program [12] detects 19 minimum possible recombination events (RM). From 0 to 8 minimum possible recombination events are calculated in the different ITS clusters (Figure 4A). No recombination was detected in *I. perado *and in clade 2 of *I. canariensis*. Clades 5 and 6 are highly recombined. An example of recombined ITS sequences of *I. canariensis *from clade 5 is shown in Figure 4B, where only informative sites are shown.

In order to exclude the possibility that the observed pattern of substitution is the result of homoplasy and to confirm that these sequences are actually recombined, maximum likelihood tests were carried out. Using PIST [13], the maximum-likelihood score of the sequences represented in Figure 4 is compared with the scores of 1000 simulated clonal sequences along the calculated maximum-likelihood tree and under the specified model of evolution (see methods). The observed score *q *(0.554) was greater than for all 1000 clonal replicates (mean value 0.381, higher value 0.505), indicating a history of recombination (significance 0.001).

The LARD maximum-likelihood method [14] was applied to find the breakpoints in the alignment, which gave the highest likelihood under an evolutionary model incorporating recombination. Only 3 sequences can be analyzed with this program. Three ITS sequences shown in Figure 4 were submitted to LARD: can 90_6_54, can 25_2_54 and can 90_4_53. Two recombination points were located by the program (at the left of positions 242 and at the left of position 582) in accordance with the delimitation indicated in Figure 4. There is no recombination point between positions 392 and 455 for these particular 3 sequences.

### Nuclear encoded plastid glutamine synthetase (nepGS) data

**Figure 5:**
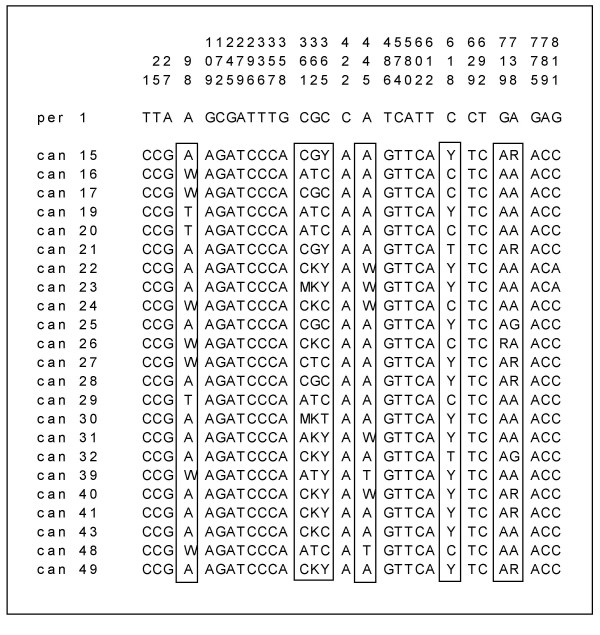
Alignment of the nuclear encoded plastid glutamine synthetase (*nepGS*) of *I. perado *and *I. canariensis*. Only variable nucleotides are represented. Polymorphic sites of *I. canariensis *are boxed. R = A or G; Y = C or T; M = A or C; M = A or C; W = A or T; K = G or T.

There is no polymorphism in *nepGS *of *I. perado*. On the other hand, *I. canariensis *shows polymorphism for this gene. Thirty sites differentiate *I. perado *from *I. canariensis*, of which eight are polymorphic in *I. canariensis*, either heterozygous or homozygous (Figure 5). For all of these eight polymorphic sites, always one of the alleles is shared with *I. perado*.

## Discussion

The high polymorphism of ITS sequences observed in *I. canariensis *is frequently reported for other plant groups [15,16]. It might have several origins: an incomplete lineage sorting from ancestral polymorphism or an horizontal transfer (introgression) through inter-specific hybridization (alloploidy), both of them not resolved by concerted evolution. Before the discussion on the origin of this polymorphism, the characterization and the fate of these different ITS sequences will be first examined.

### The genome of Ilex canariensis contains ITS pseudogenes

High polymorphism of ITS has been explained by the presence of divergent pseudogenes in *Gossypium, Nicotiana, Tripsacum, Exospermum, Zygogonum*, *Zea *[10], *Quercus *[11], *Leucaena *[17], *Adinauclea, Haldina, Mitragyna *[18] and others. Thus, this could also be the case for *I. canariensis*. Individual criteria are not sufficient to identify pseudogenes unambiguously [17] and different criteria were chosen: GC content, secondary structure of ITS 2, rate of substitution, pattern of substitution at methylated cytosine sites and substitutions at highly conserved sites of the 5.8S rDNA. ITS sequences with a GC content of 45% are unambiguously pseudogenes and satisfy to all other criteria. Moreover they have a large deletion in the ITS 1 region, which make these sequences certainly non-functional. The deletion allows an easy detection of this pseudogene on agarose gels and it is observed in many individuals of *I. canariensis *(Figure 2).

Other classes of ITS sequences with a GC content of 53–54% are also suspected to be pseudogenes by one or the other criteria but not by all of them, as expected regarding their relatively high GC content. For instance, some ITS 2 sequences of the GC 53–54% class still have a typical angiosperm secondary structure (for instance can_39_3_53, Figure 3), but have (1) an increased rate of nucleotide substitution, (2) deamination-like substitutions or (3) mutations at normally highly conserved 5.8S rDNA sites. Only the GC 61 % clade contains functional ITS sequences. Thus, it can be considered that the functional ITS GC content is 57 % for *I. perado *and above 60 %. for *I. canariensis*.

It is interesting to note that most *I. canariensis *individuals of the GC 61 % clade never have ITS pseudogenes in the GC 45 % or GC 53–54 % clades. This is probably because these individuals do not contain pseudogenes. For other individuals, a PCR selection for pseudogenes occurred, as reported for *Nicotiana *[10], in which ITS sequences with a weak secondary structure (pseudogenes) are preferentially used as templates. The inclusion of dimethylsulfoxide (DMSO) in PCR reactions [10,19], but see [18], would allow amplification of functional ITS sequences in these individuals of *I. canariensis*.

In conclusion, the high divergence found in ITS sequences of *I. canariensis *with a GC content lower than 60% (clades 1, 2, 3, 4, 5 and 6) could be explained by a release of evolutionary constraint and a subsequent high rate of substitution. Indeed, ITS sequences have functional constraints in relation with the processing of the rRNA precursor producing the functional 18S, 26S and 5.8S subunits.

### ITS sequences of Ilex canariensis are recombined

Evidence for recombination in divergent sequences is not obvious. It is difficult to recognize homoplasy generated by recombination from actual homoplasy (parallel history). Statistical methods (based on linkage desequilibrium, neutrality tests and substitution distribution along the locus) are still too rudimentary to precisely describe the recombination events in the set of ITS sequences found in *I. canariensis*. Moreover, recombinants could result from "jumping" PCR reaction [20-23], where prematurely terminated extension products can act as primer on paralogous templates. This has been shown on *nepGS *for *Oxalis *[24] and on four low-copy genes for *Gossypium *[25].

The minimum number of recombination events (RM) calculated with DnaSP [12] underestimates the total number of recombination events [26]. Thus, there is no doubt that *I. canariensis *ITS sequences experienced intra-molecular recombinations (Figure 4). The factor RM has been also calculated for PCR products of each individual in order to detect possible jumping PCR artifacts. In few of them (specimens 20, 22, 24, 27, 28 and 38) recombinants have been detected in ITS sequences resulting from a unique PCR reaction (data not shown). This could be the result of jumping PCR. However most of them are multiple recombinants and not simple recombinants as it is expected in jumping PCR [10]. As an example, the alignment represented in Figure 4 shows that specimen 90 comprises two different recombined ITS sequences resulting from the same PCR reaction, that could be the result of jumping PCR. DnaSP did not detect recombination between the four cloned ITS sequences of individual 90 because recombined fragments are paralogous sequences fragments found in other individuals. Moreover, the recombinants result from at least three crossover events and are suggested not PCR artifact. Thus, they represent true organismal intra-molecular recombinations.

The distribution of informative characters shown in Figure 4, as well as the use of programs PIST and LARD based on maximum-likelihood analyses, demonstrate unambiguously that sequences of clade 5 (Figure 4) experienced recombination events. This can not be generalized for other clades. Although DnaSP suggests recombination, an alignment demonstrating recombination, as for clade 5, was not possible for other clades, even with the help of PIST and LARD. This could be explained by the recent origin of the recombination events observed in clade 5 and by the fact that mutations did not yet obscured the recombined orthologous fragments. In this respect it is to be noticed that clade 5 shows much longer branches than other clades. This may indicate that, in clades with relatively shorter branches, mutations (or concerted evolution) did homogenize the recombined fragments, mimicking clonal divergence. Thus it can be considered that most clades also comprise recombined ITS sequences, as DnaSP suggests, but of more ancient origin than those of clade 5, and homogenized by mutation or concerted evolution.

Recombination in highly polymorphic ITS sequences seems a rule in plants. This is not surprising because the mechanisms of concerted evolution in rDNA arrays are based on crossing-over and gene conversion. It has been reported in *Begonia *[27], *Microseris *[28], *Quercus *[11], *Amelanchier *[29], *Paeonia *[30], *Buddia*, *Gossypium*, *Nicotiana*, *Tripsacum *[10], *Armeria *[31] and others.

In addition to the high rate of substitution of pseudogenes, at least some ITS sequences experienced recombination. This explains why the divergence between ITS sequences of *I. canariensis *is much higher than between ITS sequences of all other species investigated, knowing that, according to their GC content (see Figure 1), they all are potentially functional. This also explains the absence of a bootstrap support for a monophyletic clade of *I. canariensis *ITS sequences because of long branch problems due to accelerated rate of substitution and more certainly to recombination.

### The origin of the ITS polymorphism in *I. canariensis*

Two evolutionary mechanisms could produce the observed ITS polymorphism: an ancestral polymorphism escaping lineage sorting or a past or recent introgression of an alien genotype escaping concerted evolution. Because of the influence of concerted evolution, ancestral polymorphism is not the most likely explanation of ITS polymorphism [31]. On the other hand, a growing number of reports shows that ITS polymorphism is attributable to interspecific hybridization, although the parents are not always identifiable [15,16].

Assuming that multiple ITS sequences found in *I. canariensis *are the result of experienced hybridization with another species, or an ancient polymorphism with incomplete sorting, the determination of the identity of the putative hybridizing species or the finding of genetic relationships of the putative polymorphism is not obvious. This is because ITS sequences enclosed in non-functional clusters have dramatically diverged from the putative functional sequences and are recombined. All available ITS sequences of 43 other species of *Ilex*, representing a good sampling of the genus [2,3] were incorporated in the phylogenetic analysis, altogether with all ITS sequences found in *I. perado *and *I. canariensis *of Tenerife (Figure 1). Most functional (above 60% GC) and non-functional (53–54% GC) ITS clades of *I. canariensis *group together but with no bootsrap support. They group with an American lineage (*I. brevicuspis, I. anomala, I. microdunta, I. integerrima, I. theezans, I. guianensis, I. brasiliensis and I. cassine*). Only the GC 45% clade does not group with the bulk of *I. canariensis *ITS sequences. Its position is not defined and varies in the vicinity of a Eurasian lineage (*I. latifolia*, *I. leucoclada*, *I. maximocziana*, *I. rugosa and I. perado*). Thus data do not support a particular relationship of most *I. canariensis *ITS pseudogenes with another *Ilex *species, except for the pseudogenes with a GC content of 45%, that are frequently observed in *I. canariensis*.

In the case of hybridization involving the island species *I. canariensis*, the most probable candidate would be the sympatric species *I. perado*. It can not be ruled out however that the distribution of *I. canariensis *was much wider in the past [32,33] and that this hybridization may have occurred with another unknown or extinct species of the Eurasian lineage represented here by *I. latifolia, I. leucoclada, I. maximocziana, I. rugosa and I. perado*. Pseudogene sequences (particularly the ITS sequences of clade GC 45%) being too divergent and of different nucleotide composition, the observed relationship of clade GC 45% with the group of species comprising *I. perado *is questionable because of possible spurious long branch attraction. However, the data of the nuclear encoded plastid glutamine synthetase (a nuclear single copy locus) are not conflicting with an introgression of *I. perado *in *I. canariensis*. All the eight polymorphic sites observed in *I. canariensis *always comprise one allele shared with *I. perado*. Another possibility is that these ITS pseudogenes represent a relictual ancestral polymorphism in the course of elimination by lineage sorting or concerted evolution. In fact ancestral polymorphism could also be the result of ancient introgressions. The data accumulated here do not allow a definitive conclusion.

If a putative cryptic hybridization between *I. perado *and *I. canariensis *is confirmed, the introgression would be unidirectional because ITS sequences of *I. perado *do not show any polymorphism. This situation is reminiscent of the unilateral hybridization observed between *Begonia formosana *and *B. aptera*, where on 60 ITS sequences analysed in natural or artificial hybrids, 58 sequences are clustering with the ovule donor *B. formosana*, and only 2 are found clustering with the pollen donor *B. aptera *[27]. Unidirectional interspecific hybridization linked to unilateral incompatibility is frequently described in plants. However, this is not the only mechanism that can explain unidirectional hybridization. The flowering time of *I. perado *precedes the one of *I. canariensis*, thus the loading of still living *I. perado *pollen grains on young effective *I. canariensis *stigmates is more favored than the contrary. Moreover, there are much more male than female *I. perado *plants in Tenerife [34,35]. These evidences could explain the proposed unidirectional introgression.

## Conclusions

This study was undertaken with the aim to study and overcome the problem of ITS polymorphism found in many species of *Ilex *[3]. Introgression [3,4] and high polymorphism [5] have already been shown in several species of *Ilex*. Thus, precautionary measures should be taken when studying nuclear ITS sequences in the genus, particularly the search for recombinant and pseudogenes. Particular PCR conditions should be used [10,19,23]. Razafimandimbison and al. [18] were however unable to find PCR conditions to amplify a functional ITS sequence in 2 species of Rubiaceae. Amplified ITS sequences should be checked for function. A measure of the GC content (above 55% for a functional ITS sequence in *Ilex *is recommended. This study will probably make phylogenetic interpretations easier and will certainly help to the understanding of the complex evolutionary history of *Ilex *[3].

## Methods

### Material

Thirteen individuals of *I. perado *ssp. *platyphylla *Webb. & Berth. and 27 individuals of *I. canariensis *Poir. were collected in Tenerife (Las Mercedez, Aqua Garcia and Aqua Mansa) and genomic DNA was extracted from dry leaves as previously reported [2].

### Sequencing

The plastid *atpB-rbcL *spacer was sequenced for the 40 specimens of Tenerife according to Cuénoud et al. [2]. In a first experiment all ITS sequences (ITS 1, 5.8S and ITS 2) were directly sequenced from the PCR fragment according to Manen et al. [3]. All individuals of *I. perado *and five individuals of *I. canariensis *produced a perfectly readable ITS sequence with no polymorphism. On the other hand, 22 individuals of *I. canariensis *produced an unreadable highly polymorphic ITS sequence. Cloning in *E. coli *was necessary and four clones per individual were sequenced. For all specimens, the nuclear encoded plastid glutamine synthetase (*nepGS*) was amplified and sequenced according to Emshwiller and Doyle [36]. Polymorphisms were also observed in most individuals of *I. canariensis*, but as indels are not involved in the polymorphism, sequences were readable and polymorphic sites were coded according to the international code for nucleotide polymorphism (see Figure 5). Sequences are deposited at GenBank (*atpB-rbcL *spacer: AJ786512-AJ786551; ITS: AJ786413-AJ786504; *nepGS*: AJ809595-AJ809628).

### Phylogenetic analysis

The ITS sequences of *I. canariensis *and *I. perado *were aligned with ITS sequences of 43 species of *Ilex *previously studied in Manen et al. [3] and *Phyllonoma *and *Helwingia*, the closest relatives of the genus *Ilex *(for the ITS matrix see additional file 1).

Plastid *atpB-rbcL *spacer and nuclear *nepGS *matrices only comprise the sequences found in Tenerife for *I. perado *and *I. canariensis*.

Maximum parsimony trees were calculated from the *atpB-rbcL *spacer matrix, the ITS matrix and the *nepGS *matrix, using PAUP 4.0b10 [37] (heuristic search, TBR branch swapping with 10 random additions of sequences, only keeping the first 100 most parsimonious trees). Bootstrap statistics of the ITS tree were calculated from 1000 replications with the same method, except that the first 10 most parsimonious trees were kept.

### Secondary structure of ITS 2

The secondary structure of ITS 2 was investigated using the minimum free-energy program Mfold [8], which has the advantage to provide sub-optimal folding. Sequences were constrained to force the pairing of the 5'-end of the 26S and the 3'-end of the 5.8S regions according to the results of Hershkovitz and Zimmer [7].

### Pattern of substitution of ITS

In order to examine the pattern of substitution of ITS sequences found in *I. perado *and *I. canariensis*, these sequences were compared with a reconstructed *Ilex *ancestral sequence. This ancestral sequence was determined by maximum likelihood [PAUP 4.0b10 with base frequencies, ti/tv ratio, proportion of invariable sites and gamma shape parameter estimated under the HKY model (Hasegawa et al. 1985) allowing for different rate of transitions and transversions as well as unequal base frequencies] using the unique maximum parsimony tree obtained from the ITS matrix of Manen et al. [3] based on 45 species of *Ilex *and *Helwingia *and *Phyllonoma *as outgroup. For comparisons, the number of substitutions (Kimura 2-parameter) was calculated from this ancestral sequence for *I. perado *and *I. canariensis *ITS sequences using PAUP.

The high frequency of deamination-like substitutions (C -> T and G -> A at CpG and CpNpG sites) is typical for pseudogenes and was also calculated from the reconstructed *Ilex *ancestral sequence for *I. perado *and *I. canariensis *ITS sequences, according to Buckler et al. [10] and Muir et al. [11]. This frequency was compared with the frequency of C -> T and G -> A substitutions at non-methylated sites.

### Substitutions at conserved sites of the ribosomal 5.8 S subunit

Based on the alignment of fifty 5.8S sequences including vertebrates, invertebrates, fungi and plants (modified from Muir et al. [11]), 59 totally conserved sites were determined. The number of substitutions observed at these invariant sites in all *I. perado *and *I. canariensis *5.8S sequences was calculated.

### Detection of recombinations

The minimum number of recombination events RM [26] was calculated using the DnaSP program [12] in the entire ITS matrix of *I. perado *and *I. canariensis *and in different ITS clusters observed in *I. canariensis*. This program is based on linkage desequilibrium, neutrality tests and substitution distribution along the locus. ITS sequences showing strong evidence of recombination, as detected by DnaSP, were submitted to PIST [13] to calculate the probability of recombination by maximum likelihood: the tree score of these sequences is compared with the tree scores of 1000 simulated clonal sequences along the specified tree under the specified model of evolution. The score of recombined sequences will tend to have larger score than the simulated clonal sequences because richer in conflicting phylogenetic information. A maximum likelihood tree of selected *I. canariensis *ITS sequences (see results) was constructed with base frequencies, ti/tv ratio, proportion of invariable sites and gamma shape parameter estimated under the HKY model [39] and these parameters were used to calculate the tree scores of simulated clonal sequences.

The recombination points of three selected sequences (see results) showing evidence of recombination were calculated by maximum likelihood using LARD [14] with the HKY model [38]. The program calculates a maximum likelihood unrooted tree of 3 sequences and searches for a tree with a better score assuming a recombination point in the input sequences. After the calculation of one recombination point, the sequence alignment was truncated at this point to search for other potential recombination points.

## Supplementary Material

Additional File 1 ITS matrix in NEXUS Click here for file
